# Molecular Characterization of *Candida auris* Isolates at a Major Tertiary Care Center in Lebanon

**DOI:** 10.3389/fmicb.2021.770635

**Published:** 2022-01-25

**Authors:** Lina Reslan, George F. Araj, Marc Finianos, Rima El Asmar, Jaroslav Hrabak, Ghassan Dbaibo, Ibrahim Bitar

**Affiliations:** ^1^American University of Beirut, Center for Infectious Diseases Research (CIDR) and WHO Collaborating Center for Reference and Research on Bacterial Pathogens, Beirut, Lebanon; ^2^Department of Pathology and Laboratory Medicine, American University of Beirut Medical Center, Beirut, Lebanon; ^3^Department of Microbiology, Faculty of Medicine and University Hospital in Plzen, Charles University, Plzeň, Czechia; ^4^Department of Pediatrics and Adolescent Medicine, Faculty of Medicine, American University of Beirut, Beirut, Lebanon

**Keywords:** *Candida auris*, whole-genome sequencing, antifungal resistance, South Asian clade, Lebanon

## Abstract

**Background:**

The globally emerging *Candida auris* pathogens poses heavy burden to the healthcare system. Their molecular analyses assist in understanding their epidemiology, dissemination, treatment, and control. This study was warranted to describe the genomic features and drug resistance profiles using whole genome sequencing (WGS) among *C*. *auris* isolates from Lebanon.

**Methods:**

A total of 28 *C*. *auris* clinical isolates, from different hospital units, were phenotypically identified by matrix-assisted laser desorption/ionization time-of-flight (MALDI-TOF) and tested for antifungal resistance using Vitek-2 system and E test. The complete genomes were determined by WGS using long reads sequencing (PacBio) to reveal the clade distribution and antifungal resistance genes.

**Results:**

*Candida auris* revealed uniform resistance to fluconazole and amphotericin B, with full susceptibility to echinocandins. Among key resistance genes studied, only two mutations were detected: Y132F in *ERG11* gene and a novel mutation, D709E, found in *CDR1* gene encoding for an ABC efflux pump. Phylogenetically, *C*. *auris* genomes belonged to South Asian clade I and showed limited genetic diversity, suggesting person to person transmission.

**Conclusion:**

This characterization of *C*. *auris* isolates from Lebanon revealed the exclusivity of clade I lineage together with uniform resistance to fluconazole and amphotericin B. The control of such highly resistant pathogen necessitates an appropriate and rapid recovery and identification to contain spread and outbreaks.

## Introduction

*Candida auris* has been an emerging fungal infection, characterized by high transmissibility, multidrug resistance, and poor outcomes. As such, it is posing serious nosocomial health concerns globally (Forsberg et al., 2019). Its potency in colonizing patients’ skin enables patient-to-patient spread, causing outbreaks in healthcare settings (Schelenz et al., 2016; Lockhart et al., 2017).

The recognition and identification of *C*. *auris* is challenging, as the isolates of this yeast can be misidentified using commonly phenotypic laboratory methods. However, its speciation can be determined by automated systems such as the matrix-assisted laser desorption/ionization time-of-flight (MALDI-TOF) and Vitek System (Rychert et al., 2018; Patel, 2019). Molecular methods based on sequencing of the D1–D2 region of the 28s rDNA or internal transcribed spacer region provide reliable confirmation of species identification (Schoch et al., 2012; Cernakova et al., 2021). Moreover, whole-genome sequencing (WGS)-based methods have increasingly been used to detect and characterize phylogeographic types and transmission dynamics for this emerging pathogen (Lockhart et al., 2017). Since its first recognition in Japan in 2009, genetically divergent lineages have been globally identified and stratified geographically into four main clades: clade I (Southern Asia), clade II (Eastern Asia), clade III (Africa), and clade IV (South America) (Chow et al., 2020). A potentially clade V has been identified in an isolate from a patient in Iran showing a difference of >200,000 single-nucleotide polymorphisms from the other clades (Chow et al., 2019).

Several studies have revealed the genetic profiles of recovered *C*. *auris* isolates from different countries worldwide (Lockhart et al., 2017; Forsberg et al., 2019; Chow et al., 2020), including few reports from different countries in our MENA region (Al Maani et al., 2019; Alfouzan et al., 2019, 2020; Almaghrabi et al., 2020; Salah et al., 2021). In Lebanon, however, only two clinical studies were reported from the American University of Beirut Medical Center (AUBMC): the first addressed the profile of *C*. *auris* infection among 14 infected cases (Allaw et al., 2021). The second was a case report on *C*. *duobushaemulonii* associated with coronavirus disease 2019 (COVID-19) disease (Awada et al., 2021). Thus, in the absence of any characterization of *C. auris* isolates in Lebanon, this study was warranted to describe the genomic features, genetic relationships, and drug resistance profiles using WGS among *C*. *auris* isolates recovered at a major tertiary care center in this country.

## Materials and Methods

### *Candida auris* Isolates Collection

*Candida auris* isolates analyzed in this study were those recovered from patient specimens submitted for fungal investigation (prior to commencing patients’ therapy) at AUBMC Clinical Microbiology Laboratory (CML), accredited by the College of American Pathologists since 2004.

A total of 28 isolates were retrieved from 21 patients by plating the specimens on Sabouraud dextrose agar (SAB) medium and incubated them at 37°C. The specimen source of these isolates were as follows: 4 isolates from peripheral blood, 1 from central line catheter, 12 from deep tracheal aspirate (DTA), 8 from urine, 2 from skin screen, and 1 from bronchioalveolar lavage (BAL).

In six patients, isolates were simultaneously recovered from different specimen sources of the same patient: blood and central line catheter from 1 patient; DTA and urine from 2 patients; DTA and skin screening from 1 patient; DTA, skin screening, and urine from 1 patient; and DTA and blood from 1 patient.

### Identification and Speciation of *Candida auris* Isolates

The recovered *Candida* species on SAB medium was submitted directly for identification or subcultured on chocolate agar medium prior to identification. The colonies of these isolates were identified by MALDI-TOF system (Bruker Daltonik, GmbH, Bremen, Germany) and by the Vitek 2 system (BioMérieux, Marcy L’Etoile, France). Species identities were confirmed by PCR following by sequencing using ITS1F/ITS4R primers for the variable internal transcribed spacers ITS1 and ITS2 regions, located between universally conserved genes 18S, 5.8S, and 28S and NL1F/NL2R primers used to detect the D1–D2 region located at the 5′ end of the gene 28S encoding for the large nuclear ribosomal subunit (Schoch et al., 2012).

### Antifungal Susceptibility Testing

The Vitek 2 system, employing the antifungal susceptibility cards (AST-YS 08), was used to determine the minimum inhibitory concentrations (MICs) of the following antifungal agents: fluconazole, voriconazole, caspofungin, micafungin, amphotericin B, and flucytosine. The E-test (AB Biodisk, Solna, Sweden) was used to determine the MICs (μg/ml) of itraconazole (strip concentration range, 0.002–32 μg/ml), using Roswell Park Memorial Institute (RPMI) 1640 media (Sigma, St. Louis, MO, United States), according to what was reported earlier from our laboratory (Araj et al., 2015).

The interpretation of the minimum inhibitory concentrations (MICs) susceptibility breakpoints (μg/ml) for *C*. *auris* were based on CDC^1^ (accessed July 7, 2021) and Clinical and Laboratory Standards Institute (CLSI) guidelines, essentially defined based on those established for closely related *Candida* species (*Candida haemuloni*) and on expert opinion. In this context, the designated resistant breakpoints are as follows: fluconazole, ≥32 μg/ml; anidulafungin, ≥4 μg/ml; caspofungin, ≥2 μg/ml; micafungin, ≥4 μg/ml; amphotericin B, ≥2 μg/ml. Voriconazole susceptibility breakpoints are not applicable and recommended to consider using fluconazole susceptibility as a surrogate susceptibility assessment. There is no published guidance about flucytosine’s breakpoint susceptibility.

### Quality Control Isolates

The quality of test performance was controlled by including the reference strains *C. albicans* (ATCC 10231), C. *parapsilosis* (ATCC 22019), and *C*. *kruseii* (ATCC 6258).

### Whole Genome Sequencing

The genomic DNA of 29 isolates (28 *C. auris* isolates and one *C. haemuloni* as outgroup) was extracted using the NucleoSpin microbial DNA kit (Macherey-Nagel, Duren, Germany). Subsequently, the extracted DNA was sheared to obtain 15-kb fragments using Hydropore long on the Megaruptor 2 (Diagnode). Express kit 2.0 (Pacific Biosciences, Menlo Park, CA, United States) was used for library preparation using the microbial multiplexing protocol according to the manufacturer’s recommendations. Library size selection was applied using the AMPure PB beads (Pacific Biosciences, Menlo Park, CA, United States) to select for fragments above 3 kb. Using the SMRT Link v9.0, HGAP4 and microbial assembly pipelines were used to assemble the sequences with a minimum seed coverage of 30, 40, and 50 depending on the coverage. The assemblies of CA3LBN and CA7LBN were annotated; gene prediction was achieved using BRAKER2 v2.1.6 pipeline on fungus mode, which combines GeneMarK-ES v4.65 and AUGUSTUS 3.4.0 for fungal gene prediction and identifying gene locations with the corresponding CDS and messenger RNA (mRNA) qualifiers (Altschul et al., 1990; Stanke et al., 2006, 2008; Ter-Hovhannisyan et al., 2008; Camacho et al., 2009; Hoff et al., 2016, 2019; Bruna et al., 2021). Interproscan 5.50-84.0 (Jones et al., 2014) was used on the COG database to create the xml file to further incorporate in the functional annotation pipeline created by the Funannotate 1.8.7 (Blachowicz et al., 2019; Vasquez-Gross et al., 2020; Smith, 2021). The pipeline starts by running HMMscan (HMMer v3.3) (hmmer.org) with default parameters on the PFAM database, then using emapper 2.1.2 based on eggnog orthology data (Huerta-Cepas et al., 2017, 2019). Sequence searches were performed using Diamond Blastp (Buchfink et al., 2015) on UniProt DB version 2021_02 and MEROPS v12.0; the resulting annotations were combined using Gene2Product v1.69, and later, Signalp 5.0 was used to predict secreted proteins (Almagro Armenteros et al., 2019). Furthermore, transfer RNA (tRNA) identification was done using ARAGORN v1.2.41 (Laslett and Canback, 2004); tRNAs identified and found to be overlapping with any CDS sequences were removed. The ribosomal RNA (rRNA) identification was done by downloading *C. auris* rRNA sequences from the National Center for Biotechnology Information (NCBI) and then by using BLAST + v2.11.0 (Camacho et al., 2009). Assemblies and annotations were assessed using BUSCO V5.2.2 (Manni et al., 2021).

### Phylogeny

The corresponding phylogenies of the 29 genomes of this study, along with all of the 80 *C. auris* sequences found in the NCBI assembly database, were performed. Briefly, the alignment of the core genome, detection of recombination events, and single nucleotide polymorphisms (SNPs) detection were performed using Parsnp v1.2, available in the Harvest suite (Treangen et al., 2014) using CA7LBN (index case), a reference genome for clustering and using CA3LBN (identified as *C. haemuloni*) as an outgroup as described elsewhere (Prakash et al., 2016). SNPs identified in local collinear blocks were subsequently used for reconstructing an approximate maximum-likelihood tree using FastTree2 (Price et al., 2010) while including the general time reversible (GTR) model of nucleotide substitution. The Shimodaira–Hasegawa test was used to assess the support for significant clustering in the observed phylogeny. The interactive tree of life or iTOL (Letunic and Bork, 2019) was used to annotate, modify, and edit the resulting phylogeny.

### Single Nucleotide Polymorphisms Detection

In this study, SNPs of the 27 *C*. *auris* genomes were compared to the SNPs of the first *C*. *auris* (CA7LBN detected in October 2020) by using snippy multicommand (snippy-base application v4.5.0) (Seemann, 2015) that generates a core genome multiple alignment against a common reference. The CA7LBN was used as a reference, since it was the index case. The pipeline detects the variants and generates single file for each isolate listing the different variations. The results were compared to detect any possible microevolution events among the genomes with respect to the time of detection.

### Molecular Detection of Antifungal Resistance Genes Mutations

The sequences of *CDR1*, *CDR2*, *ERG1*, *ERG2*, *ERG3*, *ERG5*, *ERG6*, *ERG11*, *ERG24*, *MDR1*, *MRR1*, *TAC1*, and *UPC2* were extracted from the genome of *C. auris* B11221 and were used as reference. *C. auris* B11221 was chosen, since it is susceptible against fluconazole and amphotericin B. Then, the reference genes were blasted against the generated assemblies from the study, and the corresponding genes were detected using BLAST + v2.11.0 (Camacho et al., 2009). Protein products were then compared, and the corresponding amino acid substitutions were detected. All mutations have been confirmed by visually inspecting the alignment of the CCS reads to the assembly.

### Data Availability

All genome assemblies have been deposited at GenBank under the following accession numbers: CP077052–CP077058, CP077045–CP077051, CP076661–CP076667, CP077038–CP077044, CP077031–CP077037, CP077024–CP077030, CP076749–CP076755, CP077017–CP077023, CP077010–CP077016, CP077003–CP077009, CP076996–CP077002, CP076989–CP076995, CP076982–CP076988, CP076975–CP076981, CP076968–CP076974, CP076961–CP076967, CP076954–CP076960, CP076947–CP076953, CP076940–CP076946, CP076933–CP076939, CP076926–CP076932, CP076919–CP076925, CP076912–CP076918, CP076905–CP076911, CP076898–CP076904, CP076891–CP076897, CP076884–CP076890, CP076877–CP076883, and CP076870–CP076876. We annotated two isolates (the index and the outgroup) with the following ascension codes CP076661–CP076667 and CP076749–CP076755 corresponding to CA3LBN and CA7LBN, respectively (Supplementary Table 1).

## Results

### Patients Demographics

The gender distribution among the 21 patients showed 12 male (57%) and 9 female (43%). The average and range of age for the male and female patients were 71 years (range, 57–80 years) and 63 years (range, 34–82 years), respectively. The distribution of patients based on their clinical hospital location were nine in intensive care unit (ICU), two in respiratory care unit (RCU), four in coronary care unit (CCU), one in neuro ICU (NICU), one in emergency department (ED), and four in medical floor.

### Antifungal Susceptibility Results of *Candida auris*

The MIC_50_/MIC_90_ and range of MICs (μg/ml) for each of the tested antifungal agents against the *C*. *auris* isolates are shown in Table 1 as follows: itraconazole, 0.25/1 (range, 0.19–1); fluconazole, ≥32/≥32 (16–32); voriconazole, 0.25/0.25 (range, 0.12–4); caspofungin, 0.25/0.25 (range, 0.25–0.25); micafungin 0.12/0.12 (range, 0.064–0.12); amphotericin B, 8/8 (range, 2–16); flucytosine 1/1 (range, 1–1).

**TABLE 1:** The antifungal susceptibility results against the 28 isolates of *C. auris*.

Antifungal agent	Susceptibility findings					
	MIC50	MIC90	MIC range	%S	%I	%R
Itraconazole	0.25	1	0.19–1	0	75	25
Fluconazole	32	≥32	16–≥32	0	0	0
Voriconazole	0.25	0.25	0.12 to 4	36	61	3
Caspofungin	0.25	0.25	0.25–0.25	100	0	0
Micafungin	0.12	0.12	0.064–0.12	100	0	0
Amphotericin B	8	8	2–16	0	0	0
Flucytosine	1	1	1–1	ND	ND	ND

Among the triazole class drugs, *C*. *auris* was considered to be uniformly resistant to fluconazole, although only 54% of the isolates showed clear resistance to fluconazole (MICs ≥ 32 μg/ml), while 46% of these isolates revealed a level close to the resistance values, an MIC of 16 μg/ml. The voriconazole rates of susceptible, intermediate, and resistant *C*. *auris* were 36, 61, and 3%, respectively, and that against itraconazole (n = 8 isolates tested) were 0, 75, and 25%, respectively.

Concerning the polyene class, the specified breakpoints of resistance (MICs ≥ 2 μg/ml) for amphotericin B was detected in 100% of the tested isolates. Extrapolating the breakpoint susceptibility reported for *C*. *auris* isolates by CDC, our results indicated uniform susceptibility (100%, MICs ≤ 4 μg/ml) of these isolates against the tested echinocandin class drugs (micafungin and caspofungin) (Table 1).

### Mutations of Drug-Resistance-Associated Genes

All strains expressed fluconazole and amphotericin B resistant phenotypes. Corresponding mutations in drug-resistance-associated genes, namely, *CDR1*, *CDR2*, *ERG1*, *ERG2*, *ERG3*, *ERG5*, *ERG6*, *ERG11*, *ERG24*, *MDR1*, *MRR1*, *TAC1*, and *UPC2*, were investigated in comparison with the reference genes in B11221 strain. Two mutations were detected: the first one in the lanosterol 14-α-demethylase-encoding gene *ERG11* (Y132F) particularly in the first “hot-spot” region located between amino-acids (105–165) and another novel mutation D709E, found in *CDR1* encoding for an ABC efflux pump.

### Phylogenetic Studies and Single Nucleotide Polymorphism Analysis

**FIGURE 1:**
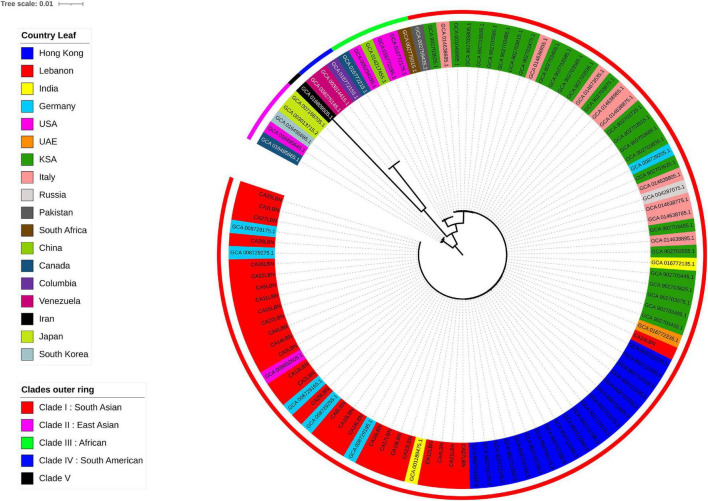
Phylogenetic tree of *C. auris* genomes, including 28 from the present study and 80 publicly available from the NCBI database. The outer most perimeter represents the five clades geographic distribution: clade I (red), clade II (purple), clade III (green), clade IV (electric blue), and clade V (black). The leaf labels indicate the strains’ ID and colored according to the geographic origin of each strain. Our 28 isolates from Lebanon fall in clade I.

The 29 genomes generated in our study, with CA3LBN (*C. Haemulonii* isolate) being the outgroup, were compared to all *C. auris* genomes available in the NCBI database (80 genomes). The results indicated that our isolates belonged to clade I (South Asian) among the five known global clades and closely clustered with the branch length showing zero differences between our isolates and the ones from United States, Germany, and India and located in close proximity to *C. auris* isolates recovered from Saudi Arabia, United Arab Emirates, and Hong Kong (Figure 1).

The genetic variations and microevolution among the 28 *C. auris* isolates were analyzed and compared to the first isolate detected in October 2020 (CA7LBN). Results revealed a range of one to six SNPs with one SNP in three isolates, two SNPs in five isolates, three SNPs in seven, four SNPs in four, five SNPs in six, and six SNPs in two isolates (Supplementary Table 1). SNPs were detected in coding and non-coding regions in the studied isolates. All isolates showed a mutation in *THR1*, a gene encoding for trihydroxynaphthalene reductase, with an amino acid substitution L188G. In addition, five other mutations were detected in different isolates as shown in Supplementary Table 2. The rest of the mutations were either in genes coding for uncharacterized proteins (hypothetical proteins) or in non-coding regions.

## Discussion

*Candida auris* infection has been tolling the healthcare systems at major hospitals globally due to its serious threatening impact and its resistance to multiple antifungal agents that limit treatment options. In Lebanon, this is the first study addressing the antifungal profile and molecular features of *C. auris* isolates using the long reads sequencing technique that generated complete genome sequences of these pathogens.

Identification of *C. auris* is generally a challenging experience. Interestingly, the first suspected encounter of *C. auris* happened when yeast colonies were recovered from the blood culture of a 34-year-old Lebanese man who works and lives in Africa (Sierra Leone) and was transferred to AUBMC for management of confirmed COVID-19 severe infection. Identification (ID) by Vitek 2 revealed *C*. *auris* (96% probability, Bionumber 4110145251301771). Repeat identification on Vitek 2 (August 12, 2020) revealed low discrimination organism (*C*. *auris* and *C*. *duobushaemuloins*; Bionumber, 4110145255321771). Unfortunately, our MALDI-TOF was affected by severe tremors of the devastating explosion of the port of Beirut (on August 4, 2020) and could not reveal any identification. Thus, discrimination between these species warranted further testing (molecular, MALDI-TOF, WGS), which was kindly extended by colleagues from Lebanon, United States, and Canada (thanked under acknowledgment), whereby all of them revealed the identification as *C*. *duobushaemulonii* (Awada et al., 2021). This reflected the difficulties in the proper identification of *C*. *auris* (Iguchi et al., 2019). Subsequently, the recovered *C*. *auris* under study were identified by the MALDI-TOF and WGS.

Concerning the phenotypic and genotypic antifungal susceptibility of *C. auris*, it also has its challenges, and it is worth to compare our findings with those reported from different parts of the world. With regard to the antifungal agents mostly used for the treatment of infection due to this pathogen, a couple of agents are relied upon including caspofungin, micafungin, fluconazole, voriconazole, and amphotericin B. Our study generally showed comparable uniform susceptible findings to those reported regionally concerning echinocandins (caspofungin and micafungin) (Emara et al., 2015; Khan et al., 2018; Al Maani et al., 2019; Almaghrabi et al., 2020). However, a sporadic resistance to echinocandins was reported from the United States (1%) (Forsberg et al., 2020), and from India and South Africa (7%) (Lockhart et al., 2017). Moreover, the uniform resistance rates to fluconazole in our study was also comparable to those reported regionally (Emara et al., 2015; Al-Siyabi et al., 2017; Mohsin et al., 2017; Salah et al., 2021) except that of one study in Oman, which reported a lower resistant rate (58%) (Al Maani et al., 2019). Globally, 93% of *C. aris* isolates were resistant to fluconazole (Lockhart et al., 2017). Concerning voriconazole, our results revealed a range of susceptibility against *C. auris*, namely, 36% S, 61% I, and 3% R, while resistant data from our region and other parts of the world showed a range of 8–73.6% (Lockhart et al., 2017; Almaghrabi et al., 2020). Nevertheless, among these triazoles, fluconazole susceptibility has been suggested by CDC to be used as a surrogate marker for second generation triazole (e.g., voriconazole) susceptibility assessment. Still, isolates that are resistant to fluconazole may respond to other triazoles occasionally (see text footnote 1).

As for amphotericin B, the resistance rate against *C. auris* in our study was uniform (100%). This resistant rate is higher than those reported rates (0–62%) from different countries in our region such as Saudi Arabia (62%) (Almaghrabi et al., 2020), Oman (33–50%) (Al-Siyabi et al., 2017; Mohsin et al., 2017; Al Maani et al., 2019), Kuwait (23%) (Emara et al., 2015), United Arab Emirates (0%) (Alatoom et al., 2018), and other countries including Pakistan and India (35%) (Lockhart et al., 2017). In the United States, the reported amphotericin B resistant rate was 33% (Forsberg et al., 2020). Fortunately, the pan- and echinocandin-resistant *C*. *auris* strains were not detected in Lebanon, as was recently reported from Texas and District of Columbia (DC) in United States (Lyman et al., 2021).

The molecular characterization of the resistance genes in our study indicated the presence of Y132F mutations in *Erg11* gene in all isolates, reflecting the resistance to azoles. These Y132F substitutions were commonly detected among South Asian isolates particularly Indian and Pakistani strains and considered clade-I-specific markers of resistance against fluconazole (Lockhart et al., 2017). Moreover, Y132F substitution in *ERG11* gene appears to be combined with a novel mutation in the *CDR1* gene, an ATP-binding cassette (ABC)-type efflux pump-encoding gene, which has previously been shown to substantially contribute to azole resistance in *C. auris* (Chowdhary et al., 2018). However, the genetic determinants promoting the increased expression of efflux pump-encoding genes in *C. auris* remain unidentified (Kim et al., 2019; Rybak et al., 2019). Rybak et al. study demonstrates that fluconazole-resistant clinical isolates of *C. auris* exhibit elevated levels of *CDR1* expression and contribute significantly to clinical resistance against the entire class of triazole antifungals. The deletion of *CDR1* in this fluconazole-resistant clinical isolate was sufficient to restore triazole resistance and increase the susceptibility of resistant strains from 64- to 128-fold. Several mechanisms have been proposed for the increased gene expression such as higher levels of mRNA stability, gene amplification, or deregulation because of point mutations in the promoter region.

Unfortunately, there is little or no information about CDR1 hotspot mutations in *C. auris* in the literature.

However, sequence analysis of the *Candida albicans CDR1* gene showed several point mutations located near the promoter of the resistant strains. These point mutations could be distributed within the recognition sequence for the binding of trans-acting transcription factors, and hence, changes to the nucleotide sequence could cause either less efficient binding of transcriptional repressors or increase in the affinity of activators to the promoter region, therefore upregulating CDR1 (Looi et al., 2005).

For amphotericin B, the 100% resistance among our isolates was detected despite the absence of main resistance drivers related to *ERG2*, *ERG3*, and *ERG6* gene mutation (Frias-De-Leon et al., 2020). Such finding indicates that the mechanism of amphotericin resistance remains to be fully elucidated.

All isolates showed a mutation in THR1 (L188G), a gene encoding a homoserine kinase involved in the biosynthesis of threonine. THR1 is considered a potential molecular target for antifungal chemotherapy, since THR1 genes are essential for growth and are required for virulence of *C. albicans* and *C. neoformans* cells (Kingsbury and McCusker, 2008). *C. albicans* cells lacking THR1 accumulate the toxic biosynthetic intermediate homoserine and are attenuated in terms of virulence and die rapidly upon threonine starvation and serum incubation (Kingsbury and McCusker, 2010a,b). Moreover, *C. albicans* THR1-depleted mutants exhibited increased sensitivity to oxidative and osmotic stress (Lee et al., 2018). Regarding the phylogenetic analysis, different findings have been reported globally. In our study, all *C*. *auris* genomes belonged to clade I showing a limited genetic diversity with SNP difference of ≤6, regardless of the recovered source, site of specimen, or time span between isolations, thereby highly reflecting an outbreak due to hospital-associated transmission and confirming what was reported earlier from the same medical center (Allaw et al., 2021). This clade I finding was similar to that reported in Qatar, Saudi Arabia, Oman, Pakistan, and India (Alfouzan et al., 2019). Other studies from different parts of the world reported other clades, for instance, clade III (Africa) among Australian isolates (Biswas et al., 2020), clade IV (South America) among isolates from Chicago (Roberts et al., 2021) and Venzuela (Lockhart et al., 2017), clade II (East Asia) from Japan, and clade V from Iran (Chow et al., 2019).

## Conclusion

This first molecular characterization of *C*. *auris* from Lebanon revealed the exclusivity of clade I lineage among the studied isolates. This clade I together with its uniform resistance to fluconazole and amphotericin B are similar to what was reported from different countries in our region. The control of such highly resistant pathogen necessitates an appropriate and rapid recovery and identification to contain spread and outbreaks.

## Data Availability Statement

The datasets presented in this study can be found in online repositories. The names of the repository/repositories and accession number(s) can be found in the article/Supplementary Material.

## Author Contributions

GA designed the study. GA and RE collected the *Candida* samples. IB, LR, MF, and RE conducted the experiments. GA, IB, LR, MF, and GD analyzed the data and wrote the manuscript. All authors revised and approved the final draft.

## Conflict of Interest

The authors declare that the research was conducted in the absence of any commercial or financial relationships that could be construed as a potential conflict of interest.

## Publisher’s Note

All claims expressed in this article are solely those of the authors and do not necessarily represent those of their affiliated organizations, or those of the publisher, the editors and the reviewers. Any product that may be evaluated in this article, or claim that may be made by its manufacturer, is not guaranteed or endorsed by the publisher.

## Footnotes

1
https://www.cdc.gov/fungal/candida-auris/c-auris-antifungal.html

## Supplementary Material

Supplementary Table 1 *Candida auris* isolates accession numbers.

Supplementary Table 2 The number and location of SNPs among *Candida auris* isolates.
